# Effects of Lipoic Acid on Immune Function, the Antioxidant Defense System, and Inflammation-Related Genes Expression of Broiler Chickens Fed Aflatoxin Contaminated Diets

**DOI:** 10.3390/ijms15045649

**Published:** 2014-04-02

**Authors:** Yan Li, Qiu-Gang Ma, Li-Hong Zhao, Hua Wei, Guo-Xiang Duan, Jian-Yun Zhang, Cheng Ji

**Affiliations:** 1 State Key Laboratory of Animal Nutrition, College of Animal Science & Technology, China Agricultural University, Beijing 100193, China; E-Mails: liyan-602@163.com (Y.L.); maqiugang@cau.edu.cn (Q.-G.M.); lihongzhao100@126.com (L.-H.Z.); duanguoxiang2014@gmail.com (G.-X.D.); futurezjy@sina.com (J.-Y.Z.); 2 Translational Medicine Lab, Chinese National Human Genome Center, Beijing 100176, China; E-Mail: huaweimda@126.com

**Keywords:** lipoic acid, aflatoxin, alleviate, inflammation, chickens

## Abstract

This study was designed to evaluate the effect of low level of Aflatoxin B_1_ (AFB_1_) on oxidative stress, immune reaction and inflammation response and the possible ameliorating effects of dietary alpha-lipoic acid (α-LA) in broilers. Birds were randomly allocated into three groups and assigned to receive different diets: basal diet, diet containing 74 μg/kg AFB_1_, and 300 mg/kg α-LA supplementation in diet containing 74 μg/kg AFB_1_ for three weeks. The results showed that the serum levels of malondialdehyde, tumor necrosis factor alpha (TNFα) and interferon gamma (IFNγ) in the AFB_1_-treated group were significantly increased than the control group. In addition, the increased expressions of interleukin 6 (*IL6*), *TNFα* and *IFNγ* were observed in birds exposed to the AFB_1_-contaminated diet. These degenerative changes were inhibited by α-LA-supplement. The activities of total superoxide dismutase and glutathione peroxidase, the levels of humoral immunity, and the expressions of nuclear factor-κB p65 and heme oxygenase-1, however, were not affected by AFB_1_. The results suggest that α-LA alleviates AFB_1_ induced oxidative stress and immune changes and modulates the inflammatory response at least partly through changes in the expression of proinflammatory cytokines of spleen such as *IL6* and *TNFα* in broiler chickens.

## 1. Introduction

Aflatoxins (AFs), produced mainly by *Aspergillus. flavus* and *A. parasiticus* [1,2] and usually found in various agricultural commodities [3], are known to be very dangerous mycotoxins. Aflatoxin B_1_ (AFB_1_), is one of the most commonly found metabolites and exhibits the highest toxigenic effects [4], which can induce reactive oxygen species (ROS) generation which causes oxidative stress, leading to impairment of DNA, RNA, proteins, lipids, and so on [5]. Meanwhile, AFB_1_ has immunosuppressive properties, and mainly exerts its effects on cell-mediated immunity while inducing an inflammatory response [3]. It is also increasingly recognized in long term consumption of low levels of AFB_1_, naturally occurring under some field conditions and in some food/feed, and can be harmful to animal and human health [6]. Furthermore, natural toxins probably pose greater threat to human and animal health than synthetic toxins [7]. However, information on the immunosuppressive effects of low dose AFB_1_ from naturally contaminated feed is limited, particularly with respect to the effects of low doses of AFB_1_ on the expression of inflammation-related genes.

Several studies have described alpha-lipoic acid (α-LA) and its reduced form dihydrolipoic acid (DHLA), as an “ideal” antioxidant couple, due to their very high protection ability against oxidative stress through multiple pathways [8,9]. Subsequent studies have shown that α-LA has been used in the prevention or treatment of several pathological conditions that are mediated via oxidative stress. Interestingly, it has been shown that α-LA can inhibit the release of various cytokines, including tumor necrosis factor alpha (TNFα) and interleukin 6 (IL6) [10]. Sola *et al.* also found that α-LA had an effect on reducing body inflammation response in the areas with metabolic syndrome [11].

Although many data on the ability of α-LA to improve antioxidant defenses and immune function and inhibit inflammatory response are available, it is not clear whether α-LA supplementation could also reverse the inflammatory status induced by AFB_1_ in broiler chickens. In this study, we investigated the effects of AFB_1_ contaminated diets on oxidative status, immunity, and the expression of inflammation-related genes of spleen in broiler chickens and whether the supplementation of α-LA is able to counteract its negative effects.

## 2. Results

### 2.1. Effect on Serum Oxidant and Antioxidant Status

**Figure 1.**
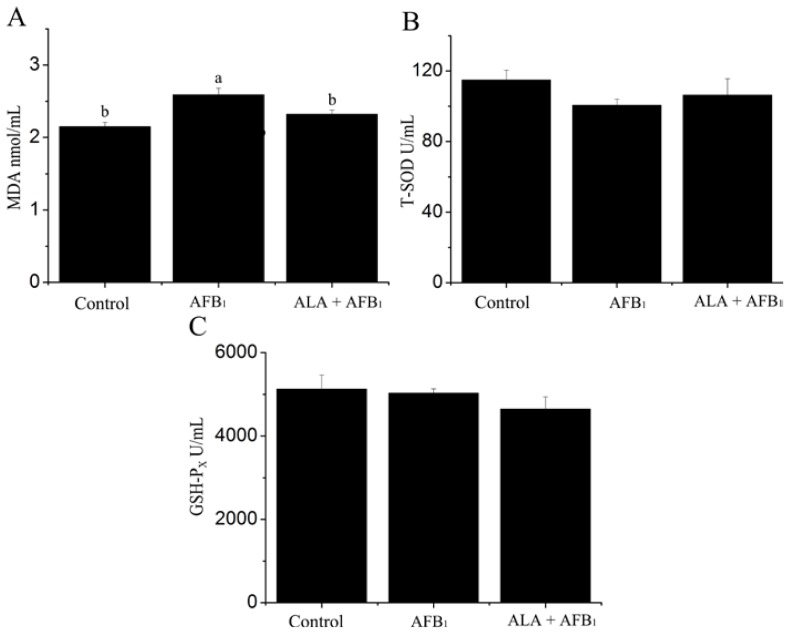
Effect of alpha-lipoic acid (α-LA) on serum antioxidant parameters in broilers fed a diet containing aflatoxin B_1_ (AFB_1_). Lipid peroxidation marker: (**A**) MDA (malondialdehyde); Antioxidant enzymes: (**B**) T-SOD (total superoxide dismutase) and (**C**) GSH-P_X_ (glutathione peroxidase). (*n* = 6). ^a,b^ Means with different letters are significantly different, *p <* 0.05.

Serum oxidant and antioxidant parameters of birds fed dietary treatments are summarized in Figure 1. The administration of AFB_1_ resulted in a significant increase in serum malondialdehyde (MDA) content when compared to untreated control group (*p <* 0.05; Figure 1A). Activities of superoxide dismutase (SOD) and glutathione peroxidase (GSH-P_X_), however, were not affected by AFB_1_ treatment (*p >* 0.05) (Figure 1B,C). Supplementation of α-LA inhibited the elevating MDA levels upon AFB_1_ administration (Figure 1A). These results indicated that the free radicals in serum were effectively scavenged when treated with α-LA.

### 2.2. Effect on Serum Immune Response

**Figure 2.**
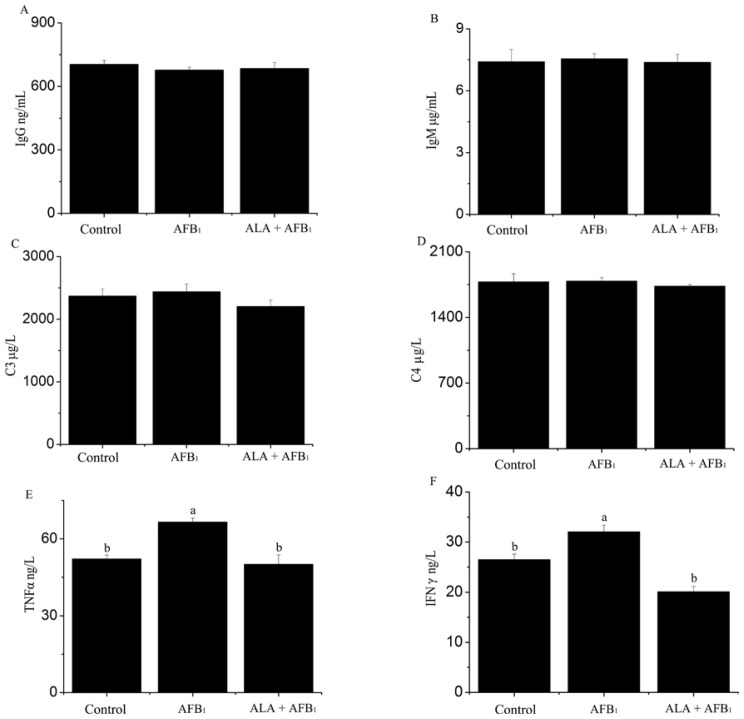
Effect of alpha-lipoic acid on serum immune response in broilers fed a diet containing aflatoxin B_1_ (AFB_1_). Humoral immunity: (**A**) IgG (immunoglobulin G); (**B**) IgM (immunoglobulin M); (**C**) C3 (complement 3); and (**D**) C4 (complement 4); Cytokines: (**E**) TNFα (tumor necrosis factor alpha) and (**F**) IFNγ (interferon gamma). (*n* = 6). ^a,b^ Means with different letters are significantly different, *p <*0.05.

Serum immune parameters of the experimental groups are summarized in Figure 2. There was a significant increase in TNFα and interferon gamma (IFNγ) in AFB_1_-treated chickens (*p <* 0.05; Figure 2E,F). However, this increase was prevented in AFB_1_-fed birds supplemented with α-LA, suggesting that α-LA supplementation can down-regulate the inflammatory processes occurring in serum of birds by AFB_1_. In addition, Figure 2A–D show that neither immunoglobulin G (IgG), immunoglobulin M (IgM), nor complement 3 (C3), complement 4 (C4) serum concentrations were affected by AFB_1_ (*p >* 0.05), suggesting that lower dosage AFB_1_ maybe not affect humoral immune response.

### 2.3. Effect on Gene mRNA Expression

**Figure 3.**
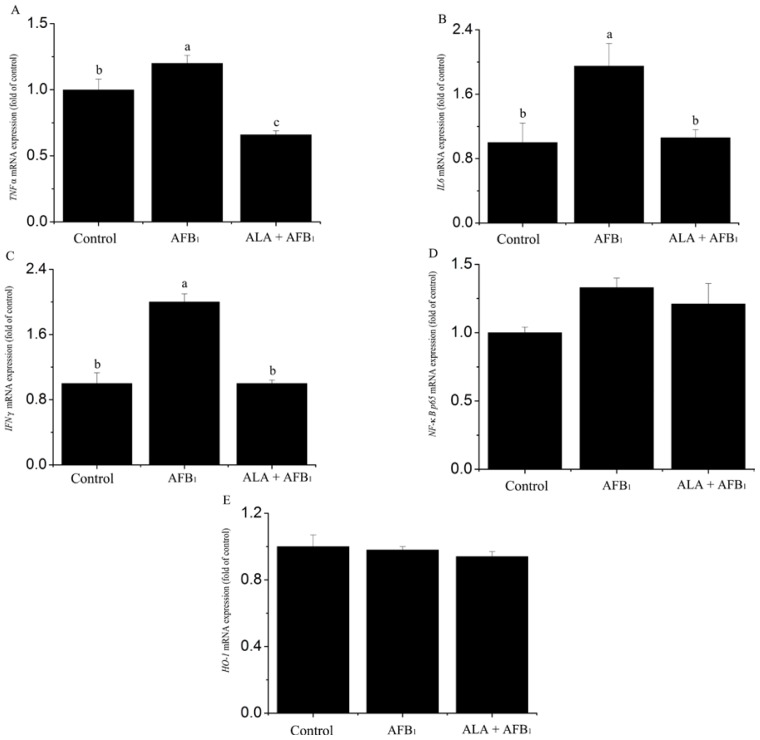
Effect of alpha-lipoic acid on inflammation-related genes of spleen in broilers fed a diet containing aflatoxin B_1_ (AFB_1_). (**A**) *TNFα* (tumor necrosis factor alpha); (**B**) *IL6* (interleukin 6); (**C**) *IFNγ* (interferon gamma); (**D**) *NF-κB* p65 (nuclear factor-κB p65); and (**E**) *HO-1* (Heme oxygenase-1). (*n* = 6). ^a,b,c^ Means with different letters are significantly different, *p <* 0.05.

The mRNA expression of inflammation-related genes was measured by real-time polymerase chain reaction (PCR) in spleen samples collected at the end of the experiment (Figure 3). In birds exposed to the AFB_1_-contaminated diet, the mRNA expression of *IL6*, *TNFα* and *IFNγ* was up-regulated (*p <* 0.05; Figure 3A–C). However, neither the gene expression of nuclear factor-κB p65 (*NF-κB p65*) nor that of heme oxygenase-1 (*HO-1*) was altered by AFB_1_ (*p >* 0.05; Figure 3D,E). Increased expression of *IL6*, *TNFα* and *IFNγ* genes due to AFB_1_ was prevented (*p <* 0.05) by the addition of α-LA to the AFB_1_ diet (Figure 3A–C).

## 3. Discussion

Previous studies have shown that acute aflatoxicosis can pose a considerable threat to productivity, tissue development and biochemical parameters [12]. Moreover, chronic AF treatment in animals can lead to poor growth performance [13], alter immune health and systemic inflammation, and cause tissue damage [14]. In this study, the effects of AFB_1_ of 74 μg/kg on productivity parameters (body weight (BW) gain, feed consumption, and feed conversion ratio) and the relative weights of spleens did not show significant changes compared to control group (*p* > 0.05; data not shown). This might be due to the low dosage of AFB_1_ used in the diet for broiler chickens. Similar results for chronic exposure to AFB_1_ have also been reported in previous studies [15,16]. It is likely that these parameters were not sufficiently sensitive to the chronic effects of low dose of AFB_1_.

One of the aims of the current study was to investigate the effects of AFB_1_ contaminated diets and the α-LA addition of feed on the serum oxidative stress. Our results showed that dietary exposure to AFB_1_ increased the serum MDA levels. The MDA level, is widely used as an indicator of lipid peroxidation (LPO), and the increase in LPO levels is associated with oxidative stress [17], which may result in pathological conditions and diseases [18]. In this study, low dose of AFB_1_ (74 μg/kg) might have resulted in overall oxidative stress in broiler chickens. It was previously reported that AFB_1_ increased the production of lipid peroxidation in broiler chickens [19] and hens [20]. The induction of oxidative stress is commonly related with an imbalance between oxidants system and antioxidants system [21]. It is of surprise that exposed AFB_1_ diets had no effect on the indicators of the enzymatic antioxidant systems of serum in chickens (*p* > 0.05). These results suggest that oxidative damage induced by AFB_1_ mainly through increasing the lipid peroxidation of serum in this model using chickens exposed to AFB_1_ contamination at a low dose of 74 μg/kg.

It is well-known that the immune system is very sensitive to AFs [22,23]. The immune system mainly involves the humoral immunity and cell-mediated immunity. In addition, there are different effects of AFB_1_ on the humoral immunity according to different species studies, but the effects are less sensitive than those on the cell-mediated immunity. It has been found that AFs can affect humoral immune [24,25]. However, in the present study, the results showed no significant changes in the serum IgG, IgA, IgM, C3 and C4 (Figure 2) in the consumption of AFB_1_ contaminated diets chickens were contradictory to the report showing that the AFB_1_-exposed diets had significant effect on the IgA and IgG titer serum concentrations of mice [26]. Similarly, some studies emphasize that AFB_1_ did not change the humoral immunity [3,27]. However, some other studies showed an increase in plasma globulin titers (IgM and IgG) in pigs exposed to dietary AFB_1_ [28]. The effects of humoral immune to AFB_1_ addition maybe depend on the type, the dose, the duration of exposure, the susceptibility of each species (pigs, rats, chicken) [29,30], or other experimental conditions. Moreover, immune response may also vary according to animal health and management status. These results confirmed that low dose AFB_1_ does not cause a significant modulation of the humoral immune response in broiler chickens.

With regard to the serum production of cytokines, which were used as further understanding of immune system status, our study found an increase of TNFα and IFNγ in broiler chickens consumed the AFB_1_ contamination diet compared to the control group. Pro-inflammatory cytokines such as TNFα and IFNγ are important in tissue immune response and inflammation. Contrary to our data, suppressive effects of inflammatory cytokines were observed in rats/mice during respiratory aflatoxicosis [31] or after oral intoxication [32]. In addition, Marin *et al.* [24] also reported that AFs could decrease proinflammatory (*IL1*, *TNF*) and increase anti-inflammatory (*IL10*) mRNA expression in phytohemagglutinin-stimulated blood cells. In agree with our study, Chaytor *et al.* [14] reported an increased serum TNFα level of broiler chickens fed 180 μg of aflatoxin (AF)/kg and 900 μg of deoxynivalenol (DON)/kg, which maybe due to an acute-phase response to inflammation induced by the toxic effects of AFs and DON. Thus, this issue is still controversial and need further investigation. These results indicate that low level AFB_1_ could lead to inflammation and alter immune response. These changes may be associated with oxidative stress by AFB_1_, which was supported by Shay *et al.* [9].

As mentioned before, part of the initial aim for the current study was to investigate the *in vivo* expression of inflammation-related genes (mainly proinflammation cytokines such as *TNFα*, *IL6*, *IFNγ* and transcription factors) in broiler chickens consumed AFB_1_ contaminated feed. Given that some cytokines (TNFα, IL6, and IFNγ) and transcription factors (NF-κB, HO-1) are critical for both inflammation response and tissue function, it has been observed that AF preforms part of its immunosuppressive effects through some cytokines [33]. Meanwhile, many researchers have reported that AFB_1_ can lead to the alteration of cytokine expression and production *in vitro* or *ex vivo* [32,34,35]. However, no data is available concerning the *in vivo* modulation of AFB_1_ on inflammation-related gene of lymphoid organs, except only one study using pigs exposed to diet contaminated with 385, 867 or 1807 μg pure AFB_1_ per kg feed. To our best knowledge, this is the first study that investigates the expression of inflammation-related genes of spleen during AFB_1_ intoxication in chickens. In the current study, the mRNA level of *TNFα*, *IL6*, *IFNγ*, *NF-κB p65* and *HO-1* in spleen were measured by quantitative PCR. Spleen was chosen because this secondary lymphoid organ is a site for both innate and adaptive immune response [3,36]. The increase of *IFNγ*, *IL6* and *TNFα* mRNA (proinflammatory cytokines) was observed in chickens exposed to AFB_1_ (Figure 3). Our data indicate that low dose of AFB_1_ also resulted in the inflammation response in spleen, and the inflammatory status was also observed in serum of chickens. This is in agreement with results obtained in pigs that received with 385, 867 or 1807 μg AFB_1_/kg feed [3], which finding is a significant up-regulation of cytokines mRNA by the highest dose of AFB_1_. Modification of inflammation-related gene expression by AFB_1_ has been confirmed in the liver of rats [37]. By using rats exposed to a chronic intermittent dosing AFB_1_ (1600 μg/kg feed), Hinton *et al.* [34] demonstrated that the induction of an inflammatory response (the increase in the production of IL1 and IL6) was associated with liver injury. In a parallel research, we also observed liver lesions of aflatoxicosis including oxidative damage and an inflammatory infiltrate in chickens exposed to AFB_1_ (74 μg/kg feed) (unpublished). So the expression of the splenic inflammatory cytokine, herein, is consistent with our observations of liver inflammation response. Moreover it supports the hypothesis that the inflammatory reaction at partly is responsible for the liver cell injury induced by AF.

It has been reported that α-LA has been used as a nutritive supplement, a pharmacotherapy and antioxidant in foods [9]. Indeed, the supplement of α-LA can protect against oxidative stress induced either by certain drugs or under various physiological and pathophysiological conditions [38,39]. In addition, previous studies undertaken in our lab have shown that supplementation of α-LA into AFs free-diets could enhance the antioxidant capability of broilers [40]. We showed that α-LA supplementation to AFB_1_-treated chickens significantly decreased the levels of MDA compared with those treated with AFB_1_ alone. This may be attributed to the ability that α-LA can inhibit lipid peroxidation by directly scavenging free radicals and chelating metals that play an important role in increased production of free radicals [8,41]. Furthermore, there is evidence that α-LA has an anti-inflammatory effect [42] by blocking the production and expression of cytokines and membrane co-stimulatory molecules. In our current study, α-LA was shown to inhibit the production of TNFα and IFNγ and the expression of the *IFNγ*, *IL6* and *TNFα* genes of spleens in AFB_1_-treated chickens. In accordance with these observations obtained herein, Zhang *et al.* [43], and Kang *et al.* [44], have demonstrated various anti-inflammatory action of α-LA. All these results also support the anti-inflammination effects of α-LA, which may be responsible for its effect of potent antioxidant.

Previous studies showed that α-LA decreased the production/expression of proinflammatory cytokines and protected from the tissue damage caused by LPS in associated with alterations of NF-κB and HO-1 activity [45,46]. It is surprise of that no change in the expression of *NF-κB* and *HO-1* gene was observed in different diet, suggesting that the suppression of α-LA on the spleen inflammation induced by low dose AFB1 might be not through modulating the transcriptional level of NF-κB and HO-1. Further studies are needed to confirm the underling mechanisms of α-LA alleviating the spleen inflammatory response caused by chronic low dose of AFB_1_.

## 4. Experimental Section

### 4.1. Collection of Feed Ingredients Contaminated with Aflatoxin B_1_

A total of 100 feed ingredients were sampled in the scope of the nation, and the contents of mycotoxins including AFB_1_, deoxynivalenol, zearalenone and ochratoxin A were tested and determined using high performance liquid chromatography (HPLC) according to the method of Trucksess *et al.* [47]. Two samples of AFB-free peanut meal and peanut meal seriously contaminated with AFB_1_ (330 μg/kg) were selected and incorporated into the basal diet by proportion.

### 4.2. Animals

One-day-old male broiler chickens (Ross 308) were obtained from a commercial hatchery (Chia Tai Co., Ltd., Baoding, Hebei, China). The brooding temperature was maintained at 35 °C (65% relative humidity (RH)) for the first 2 days, and then decreased gradually to 21 °C (45% RH) until 28 days and maintained as such until the end of the experiment. The light regime was 23l:1d. All chicks were provided ad libitum to water and a commercial diet during the rearing period. The animal care protocol in this experiment was according to commercial management practice, and approved by the Animal Welfare Committee of China Agricultural University.

### 4.3. Experimental Design

After a 10 days adaptation period to the diet and surrounding, a total of 120 eleven-day-old birds with similar body weights (BW) were randomly assigned to 3 groups with 4 replicates pens of 10 birds per pen. Three treatment groups included: fed the basal diet with 21% normal peanut meal (without any mycotoxin) (control); fed the diet containing 74 μg/kg AFB_1_ (21% moldy peanut meal naturally contaminated with 330 μg/kg AFB_1_ substituted for normal peanut meal by the same proportion in basal diet); fed the diet supplemented with 300 mg/kg dl-α-lipoic acid (Sigma Chemical, St. Louis, MO, USA) and AFB_1_ (determined 74 μg/kg AFB_1_ without other mycotoxins). All essential nutrients in the basal diet met or were slightly lower than the nutrient requirements of National Research Council [48]. The feeding trial period lasted for 3 weeks.

At the end of the experiment, six chicks with body weights close to the average were selected from per treatment. Blood sample was drawn from a wing vein with a 5 mL syringe within 30 s and then transferred to iced tubes. Serum was obtained from the blood by centrifugation at 3000× *g* for 10 min and was stored at –20 °C for further biochemical analysis. All chickens were sacrificed for tissue collection after blood sample collection [49]. A portion of the spleen was collected from the animals, flash-frozen in liquid nitrogen and stored at –70 °C until processed for inflammation-related genes mRNA measurements.

### 4.4. Parameter Analysis

Lipid peroxidation was assessed on the basic of serum MDA content and antioxidant enzyme levels in serum were estimated by measuring SOD and GSH-P_X_ activities. The analyses were performed using MDA, SOD and GSH-P_X_ Assay Kits, which were obtained from the Nanjing Jian-cheng Bioengineering Institute (Nanjing, Jiangsu, China). Serum MDA content was measured using the thiobarbituric acid method [50], reading the absorbance at 532 nm with the spectrometer and were expressed in nmol/mL. Serum SOD activity was assayed by the xanthine oxidase method [51], which monitor the degree of inhibition of nitroblue tetrazolium reduction by O_2_-generated by xanthine and xanthine oxidase, the absorbance was read at 550 nm using a spectrophotometer. Serum GSH-P_X_ activity was detected by determination of the reduction of glutathione (GSH), the GSH react with 5,5-dithiobis (2-nitrobenzoic acid), produce yellow colored compounds which were detected at 412 nm using a spectrophotometer [52].

In this present study, immune response status in serum was estimated by measuring the levels of IgG, IgM, C3, C4, TNFα and IFNγ. These indices were analyzed using ELISA method. The details of all determination procedures followed the manufacturer’s instructions for the commercial kits (R&D System, Inc., Minneapolis, MN, USA).

### 4.5. Gene Expression Analyses

The mRNA concentrations of spleens for broiler chickens *TNFα*, *IL6*, *IFNγ*, *NF-κB p65*, and *HO-1* were quantified by quantitative real time PCR. *β-actin* was used as a house-keeping gene to normalize the gene expression data. The primer information for all the genes is listed in Table 1.

Total RNAs were extracted from the spleens by TRIZOL Reagent Kit (Invitrogen, San Diego, CA, USA). Reverse transcription (RT) was carried out using an RT reactions (10 μL) consisted of 500 ng total RNA, 5 mmol/L MgCl_2_, 1 μL RT buffer, 1 mmol/L dNTP, 2.5 U AMV, 0.7 nmol/L oligo d(T) and 10 U ribonuclease inhibitor (TaKaRa, Dalian, China). The cDNA was amplified in a 20 μL PCR reaction containing 0.2 μmol/L of each specific primer (Sangon, Shanghai, China) and SYBR green master mix (TaKaRa, Dalian, China). Each cycle consisted of denaturation at 95 °C for 10 s, annealing at 95 °C for 5 s, and extension at 60 °C for 34 s. Each sample was measured in duplicate analysis. If the difference between two duplications was greater than 15%, the sample was analyzed again. The PCR products were verified by electrophoresis on a 0.8% agarose-gel and by DNA sequencing. Standard curves were generated using pooled cDNA from the samples being assayed, and the comparative cycle threshold (*C*_t_) method (2^−ΔΔ^*^C^*^t^) was used to quantitate mRNA expression according to Livak and Schmittgen [55].

### 4.6. Statistical Analyses

The variability of results was expressed as the mean ± standard error (*X* ± SE). The significance of differences between mean values was determined using one-way ANOVA. Means were considered significantly different at *p <* 0.05.

## 5. Conclusions

Based on our results and the aforementioned discussion, we showed that the production of cytokines and spleen inflammation response caused by consuming diets containing AFB_1_ at concentrations as low as 74 μg/kg. The supplement of α-LA alleviates oxidative stress and immune changes induced by AFB_1_, and modulates the inflammatory response at least partly through changes in the expression of proinflammatory cytokines of spleen in broiler chickens. Thus, α-LA may be the possible potential application by feed to counteract some of the negative effects of AFB_1_ in poultry. Moreover, this study data may also provide some new insights for prevention and treatment of poison/toxicity in human and animal.

## Figures and Tables

**Table 1.** Gene-specific primer of related genes.

Gene	Genebank number	Primers position	Primers sequences(5′→3′)	Product size
*β-actin*	AW05994	Forward	tgcgtgacatcaaggagaag	300 bp
		Reverse	tgccagggtacattgtggta	
*TNFα* #	AY765397.1	Forward	tgtgtatgtgcagcaacccgtagt	229 bp
		Reverse	ggcattgcaatttggacagaagt	
*IFNγ*	NM_205149.1	Forward	tgagccagattgtttcgatg	246 bp
		Reverse	tccttttgaaactcggagga	
*IL6*	NM_204628.1	Forward	agatgtgcaagaagttcacc	286 bp
		Reverse	accacttcatcgggatttat	
*NF-κB p65*	D13719.1	Forward	ttgctgctggagttgatgtc	167 bp
		Reverse	tgctatgtgaagaggcgttg	
*HO-1* *	NM_205344.1	Forward	ggtcccgaatgaatgcccttg	137 bp
		Reverse	accgttctcctggctcttgg	

# From Hong *et al.* [53]; and

* from Druyan *et al.* [54].
